# Supplementary material for: Exploring the Biocontrol Potential of Phanerochaete chrysosporium against Wheat Crown Rot
Source: J Fungi (Basel). 2024 Sep 7;10(9):641. doi: 10.3390/jof10090641 (PMC11432967; doi:10.3390/jof10090641)
Supplement: Supplementary file 1 [file jof-10-00641-s001.zip › jof-3119978-supplementary.pdf]

Exploring the Biocontrol Potential of *Phanerochaete chrysosporium* Against Wheat Crown Rot

Table S1 Primer pairs of genes for qPCR to validate transcriptome

Gene	Forward Primer（5′-3′）	Reverse Primer（5′-3′）
gpd	ATGTTTGTCTGCGGTGTCA	TTCTGGGTAGCGGTGGTAG
AGR57_4555	CGCTCAAAGCCAACTTCCTC	CGCGATTGACTCCAGCTACTT
AGR57_10505	CGGGCTCTGTCGGCTTTA	GCGATTGGATGCGGTTGAT
AGR57_7806	GACCACCCAGCAGTCGTTC	TGTAGATGTTGATGAGGATACCG
AGR57_3705	TTCGGGCTTCGCAGATGA	CGTAGTCCGCAAAGTCCTGGTT
AGR57_9511	TCGCCGACGACAAATCA	TTCCACCACGAATCTTGAGT
AGR57_8924	ACTTCGCAGCACCACCCT	TTCGTCCTGGCAGAACACC
AGR57_8923	GTTCAGCGAAGAGCACTACCGA	TTCACTCCGAGGGCACCAC
AGR57_3689	CACGCCCGAAGGCAAGAT	AGGAGGCTGAGGCCGAAGA
AGR57_4271	CGACCCTCGTGATGATTGC	CGTCCCACCGATGCTGAA

Table S2 Primer pairs of plant antioxidant enzyme and defense-related genes

Gene	Forward Primer（5′-3′）	Reverse Primer（5′-3′）
SOD	GAAGAACCTCAAGCCTATCAGCG	CAGAGGGTGCTTTACAAGGATCT
POD	GCCGTTGAGATTACTGGTGGAC	GTCTTCCTGATGCTACCAAGGG
PR2	ACTTTTCAGAGGAAGCAGCGA	AGTCTGCCGATGGTATACACAA
PR 1-2	GCAAAGGTGACTGCCAACTG	AAGTGGCACAGATGCAGTGA
PP4	CGCCCAAGGACATCCTGATT	ACGCTTTATTCGCGGTTTCG
α- Tubulin	AAAGGAGGATGCGGCGAACAA	AGTGTTGATGAGGCGGCTTGT
